# Effectiveness of prepregnancy care for women with pregestational diabetes mellitus: protocol for a systematic review of the literature and identification of a core outcomes set using a Delphi survey

**DOI:** 10.1186/s13063-015-0894-8

**Published:** 2015-08-14

**Authors:** Aoife M. Egan, Valerie Smith, Declan Devane, Fidelma P. Dunne

**Affiliations:** Galway Diabetes Research Centre, School of Medicine, National University of Ireland, Galway, Ireland; School of Nursing & Midwifery, Trinity College Dublin, Dublin, Ireland; School of Nursing & Midwifery, National University of Ireland, Galway, Ireland; Saolta University Health Care Group, Galway, Ireland; Health Research Board – Trials Methodology Research Network (HRB-TMRN), Galway, Ireland

**Keywords:** Consensus, Core outcome set, Delphi, Pregestational diabetes, Prepregnancy care, Type 1 diabetes, Type 2 diabetes

## Abstract

**Background:**

Women with pregnancy complicated by pregestational diabetes experience increased rates of adverse pregnancy outcomes. Prepregnancy care is the targeted support and additional care offered to those women who are planning pregnancy and is associated with improved outcomes. However, there is significant heterogeneity in the outcomes measured and reported in studies evaluating the effects of prepregnancy care, which makes meaningful comparison difficult. The aim of this article is to present a protocol for a study to develop a Core Outcome Set (COS) for trials and other studies evaluating the effectiveness of prepregnancy care for women with pregestational diabetes mellitus.

**Methods/Design:**

This study will include a systematic review of the literature to identify outcomes that have previously been reported in studies evaluating prepregnancy care for women with pregestational diabetes. We will then prioritise these outcomes from the perspective of key stakeholders, including women with pregestational diabetes as well as clinicians, using a Delphi survey. A final consensus meeting will be held with stakeholders to review and finalise the outcomes.

**Discussion:**

The expectation is that the COS will always be collected and reported in all clinical trials, audits of practice and other forms of research that involve prepregnancy care programs for women with pregestational diabetes. This will facilitate comparing and contrasting of studies and allow for combining of appropriate studies with the ultimate goal of improved patient care.

**Electronic supplementary material:**

The online version of this article (doi:10.1186/s13063-015-0894-8) contains supplementary material, which is available to authorized users.

## Background

Despite modern approaches to diabetes care, pregnancy can pose significant risk to both mother and infant in the presence of type 1 or type 2 diabetes, also described as pregestational diabetes [1]. Women with pregnancy complicated by diabetes have increased rates of adverse pregnancy outcomes, including a congenital malformation rate twice that of the general maternity population, a fivefold increased risk of stillbirth and a threefold increased risk of perinatal mortality [2, 3]. The caesarean section rate for women with diabetes in pregnancy is 67 %, as compared with 22 % in the background population. The rate of macrosomia among with diabetes in pregnancy is approximately 21 %, as compared with 11 % in the general maternity population [2–5].

The provision of structured clinical care before pregnancy, however, is associated with improved outcomes [6, 7]. This is particularly true for rates of malformations and perinatal mortality, as these are related to a greater extent to poor glycaemic control earlier rather than later in pregnancy [8]. The mechanism of this relationship is clear when one considers that foetal organogenesis is essentially completed by 7 weeks of gestation, often before the woman knows she is pregnant [9]. It is recommended, therefore, that all women of reproductive age with diabetes be offered annual preconception counselling and advised to avoid unplanned pregnancy [6, 10]. Prepregnancy care is the targeted support, and additional care is offered to women who are actually planning pregnancy [6]. Once women are ready to engage with prepregnancy care, they are reviewed at intervals of 1–3 months by a multidisciplinary team. This team typically includes a diabetes physician, a diabetes nurse and/or midwife specialist and a dietician. Women will undergo a full medication review, a review of diabetes-related complications, an assessment for other conditions more commonly associated with diabetes (e.g., thyroid and coeliac disease) and commence folic acid therapy. Diabetes-related complications and thyroid status will be assessed, and glycaemic control will be optimized. It is recommended that the glycosylated haemoglobin A1c (HbA1c) level should be below 48 mmol/mol (6.5 %) if achievable without causing problematic hypoglycaemia [10]. There is not, however, an agreed standardised approach for provision of this care, and, though many groups have reported positive benefits associated with their own prepregnancy programs, outcomes reported are varied and diverse [6, 7, 11].

### Development of a core outcome set

Clinical studies should have defined outcomes that answer questions generated by the main hypothesis [12]. The outcomes potentially relevant to prepregnancy care programs are diverse and include both maternal and foetal outcomes. Unfortunately, there is significant heterogeneity in the outcomes measured and reported in studies evaluating the effects of prepregnancy care for women with pregestational diabetes. This is highlighted in a systematic review and meta-analysis by Wahabi et al., who noted that, of 21 included studies, only 13 included congenital malformations as an outcome of prepregnancy care and only 5 included the difference in the level of HbA1c [13]. This inconsistency in outcomes between studies makes meaningful comparison difficult and limits the ability to combine individual studies’ findings into summarised estimates. Further concerns include non-uniform outcome selection and reporting bias in clinical studies. These issues involve researchers’ selecting outcomes that suit their needs and reporting only favourable results [14, 15]. To further highlight this issue, a recent descriptive survey of 6127 outcomes in 788 Cochrane reviews found that 37 % of prespecified outcomes were not actually reported [16]. If study findings are to influence policy and practice, then the chosen outcomes need to be relevant and important to key stakeholders to facilitate development of high-quality care [15]. The authors of a recent Cochrane review [17] could identify only one randomised controlled trial evaluating different models of prepregnancy care for women with diabetes [18]. They recommended strongly the need for further, large, high-quality randomised controlled trials to evaluate the effect of different protocols of preconception care for women with preexisting diabetes [17]. There is a risk that, owing to lack of guidance on appropriate outcomes, the usefulness of this future research in informing clinical practice will be limited.

One method of addressing this lack of uniformity is through the development, use and reporting of an agreed set of outcomes, known as a Core Outcome Set (COS). The expectation is that the COS will always be collected and reported in all clinical trials, audits of practice and other forms of research that involve a specific clinical condition [15]. This will facilitate comparing and contrasting of studies and allow for combining of appropriate studies. The development of such COS is facilitated and supported by the Core Outcome Measures in Effectiveness Trials Initiative, which was launched in 2010 and brings together interested researchers while minimising duplication of effort [15, 19]. Importantly, a COS does not restrict researchers from evaluating additional outcomes; rather, it represents a minimum that should be collected and reported. COS are now developed for many aspects of patient care. In 2014, the editors of over 50 journals began the Core Outcomes in Women’s Health Initiative [20–23]. This initiative has a number of aims, which include encouraging researchers to develop COS in the field of women’s health, organising robust peer review and facilitating effective dissemination of manuscripts [20].

### Study aim

The aim of this article is to present a protocol for a study to develop COS for trials and other studies evaluating the effectiveness of prepregnancy care for women with pregestational diabetes mellitus.

### Study objectives

Our objective is to describe in detail the following steps necessary to develop the COS:

1. Complete a systematic literature review to identify outcomes reported in studies evaluating prepregnancy care for women with pregestational diabetes
2. Prioritise these outcomes from the perspective of key stakeholders, including women with pregestational diabetes and clinicians, using a Delphi survey and consensus meeting

## Methods/Design

### Ethics

Ethical approval to conduct this study was sought and obtained from the ethics committee at Galway University Hospitals (home institution of the study’s principal investigator).

### Part 1: Generation of a list containing possible relevant outcomes

#### Systematic review question

What are the outcomes reported in studies assessing the effectiveness of prepregnancy care for women with pregestational diabetes?

#### Methods

Using a comprehensive search strategy, the following databases will be searched for relevant studies: MEDLINE), Embase, Web of Science, Cochrane Library and CINAHL. ClinicalTrials.gov will also be searched for relevant, ongoing trials. Key terms used to guide the search will include ‘diabetes’, ‘pregnancy’, ‘prepregnancy’, ‘pre-pregnancy’, ‘preconception’ and ‘preconception’, combined as appropriate using the Boolean operands ‘AND’ and ‘OR’. Additional file 1 outlines the detailed search strategies and time limits applied to each of the databases. The reference lists of all relevant studies will be searched for additional relevant studies not retrieved from the electronic database search. Language restrictions will not be applied to the search strategy; however, selection of relevant articles will be restricted to English-language publications. Searching all languages will enable us to identify the extent of potentially eligible additional studies that will not be included and consider if this presents a source of language bias.

#### Types of studies

For the purpose of this phase of the development of the COS, we will include prospective cohort studies, case–control trials, randomised trials and systematic reviews (with and without meta-analyses) comparing women who did and did not receive prepregnancy care. In line with prior work in this area, we will exclude review reports and reports of conference proceedings or abstracts when there is no complete description of the trial or study [13].

#### Types of interventions

We will use a definition of prepregnancy care similar to that of Wahabi and colleagues [13]. This includes the following as a sole intervention or in combination for women with type 1 and/or type 2 diabetes:

1. Glycaemic control by insulin, oral pharmacologic agents and/or diet, aiming at fasting blood glucose ≤5.7 mmol/L and/or postprandial blood glucose ≤7.8 mmol/L and/or HbA1c ≤7.0 %
2. Counselling and/or education about diabetes complications during pregnancy, the importance of glycaemic control and self-monitoring of blood glucose level
3. Prepregnancy screening and treatment of complications of diabetes (e.g., retinopathy, nephropathy, hypertension)
4. The use of contraception until optimization of glycaemic control is achieved
5. Vitamin or folic acid supplementation in the prepregnancy period

#### Types of participants

Participants will be women of reproductive age with pregestational diabetes mellitus who were not pregnant at the time of intervention.

#### Study assessment

An initial selection of studies identified in the search will be performed using the predetermined review inclusion criteria. Two reviewers (FPD and AME) will independently assess the titles and abstracts of selected studies. Full texts of studies meeting the inclusion criteria, or where there is uncertainty regarding inclusion at title and abstract screening, will be retrieved and reviewed with final decisions on inclusion or exclusion achieved through consensus. In cases of disagreement, a third independent reviewer (VS) will be consulted.

#### Data extraction

The following data will be extracted from each study: study design, author details, year and journal of publication, targeted condition (i.e., type 1 or type 2 diabetes or both), interventions under investigation, each effectiveness outcome specified in Methods section and/or findings, whether the outcome was defined or not, the definition used, the indicators and/or tools used to measure the outcomes and the time points or periods of outcome measurement. Two review authors (AME and FPD) will extract data independently, review the data together, assess consensus and ensure that all outcomes have been identified. Disagreement will be resolved through discussion. Where a resolution is not possible, a third reviewer (VS) will be consulted.

#### Data analysis and presentation

Data will be tabulated so that each study is listed and all outcomes measured in each study are displayed separately. Following this, outcomes will be further grouped under domains (e.g., maternal outcomes and neonatal outcomes) following a review of the outcomes by AME, FPD and DD. The number of outcomes used to reflect each domain and the number of different definitions and methods of measurements used will be described.

### Part 2: Developing a consensus on outcomes important to different stakeholders using a Delphi survey and consensus meeting

#### Delphi survey

##### Methods

To develop consensus on outcomes of importance to key stakeholders for inclusion in the COS, a Delphi method will be adopted. This method is iterative and uses a series of rounds of data collection and analysis to condense the opinions of individuals into a group consensus. Typically, it involves the use of sequential rounds of questionnaires designed to elicit participants’ opinions on a particular topic. Responses to each round are collated, analysed and redistributed to participants for further comment in successive rounds [21]. This technique has advantages over less structured methods of reaching consensus, such as roundtable discussions. It avoids a situation where certain individuals can dominate a discussion or where other individuals feel obliged to agree with the opinions of more senior members [24]. It also facilitates wide international participation and increased numbers of stakeholders informing the prioritisation process. To improve efficiency, the questionnaire will be completed online using QuestionPro software (http://www.questionpro.com/) or similar such software appropriate to online survey design.

The list of potential outcomes finalised in part 1 will be formatted into a list of outcomes with a response designed to allow the participants to rate each one of the outcomes on a 9-point Likert-type scale, with higher values representing increased importance for inclusion of the outcome in the COS. Following review of the outcome list by the research group (AME, VS, DD and FPD), the list will be circulated to the study advisory group (SAG). The SAG, composed of key stakeholders, will be asked to comment on the overall list of outcomes and the suitability of the domains under which they are grouped. They will additionally be asked to list any other outcomes that they think should be included. These other outcomes will be added to the list for inclusion in the Delphi survey. Members of the SAG will not participate in the Delphi exercise, as they had a role in study design, which may influence scoring. Instead, they will be invited to participate in the final consensus meeting.

#### Participants

The key stakeholders include women, clinicians and researchers. Women, who represent service users, will have a known diagnosis of type 1 or type 2 diabetes mellitus or represent women with such diagnoses. Recruitment will include listed groups of diabetes services users (e.g., Diabetes Ireland) accessed via the electronic discussion email list manager. The manager will be emailed information on the survey and a request to distribute an invitation email to members on their email lists. The list managers will have an opportunity to contact the researcher directly to clarify any issues or to seek further information about the survey and the research before making a decision. The distribution of the survey will be at the discretion of the email list manager. Clinical leads at participating hospitals will also be asked to invite service users to participate via their prepregnancy and diabetes care clinical teams. Clinicians will include obstetricians, endocrinologists, midwives, diabetes midwife and/or nurse specialists and dieticians. Researchers with expertise in diabetes care will be also invited to participate. Clinicians and researchers will be invited from specialist centres globally. Clinical leads will be identified and recruited through the Diabetes in Pregnancy Study Group, which is a study group of the European Association for the Study of Diabetes. These clinical leads will be invited to participate by email and will be asked to forward the study details to other members of their prepregnancy care teams. Snowball sampling will be used, whereby participants from all the above groups will be asked to forward the invitation to others whom they regard as having the required expertise. Although there is no consensus on the ideal number of participants for a Delphi survey, we will aim for a total of 180 participants, including at least 30 service users [21, 25]. Each participant will be emailed a letter of invitation outlining the study and the link to the online survey. Informed consent to participate in the study will be obtained from each participant upon registration for the online Delphi questionnaire. The consent process will occur before submission of any answers. The importance of completing the whole Delphi survey process will be emphasised, and generic reminder emails will be sent to aid completion of each round. A unique identifier will be assigned to each participant, tracked to their email address, which will allow monitoring of attrition at each round.

#### Delphi study round 1

The online questionnaire will request the participant’s name, email address and centre with which they are aligned in either receiving (users) or providing and/or researching on prepregnancy diabetes care (clinicians and/or researchers). Participants will be asked to identify the stakeholder group and subgroup to which they belong. They will be asked to complete the questionnaire within 3 weeks and will be prompted after week 2 with an email reminder. The questionnaires will contain lay terminology alongside clinical terms to assist women in understanding complex terminology.

The list of outcomes to be scored will be ordered alphabetically to avoid weighting of outcomes caused by the order in which they are displayed. There will be an option for a participant to add up to two additional outcomes and provide an associated score. They will be asked the following key question: ‘Prepregnancy care for women with pre-existing diabetes can have a variety of beneficial effects, each of which could be measured as an outcome in a clinical study. Please score how important each of the following outcomes is on a scale of 1–9’. Scores will be grouped into 1–3 = not important, 4–6 = important but not critical or 7–9 = critical. This scale was devised by the Grading of Recommendations Assessment, Development and Evaluation and has been adopted in other core outcome development research groups using the Delphi method [12, 25].

#### Analysis of round 1

The results of round 1 will be summarised using descriptive statistics, including the proportion of participants scoring for each rating point on the Likert scale (i.e., for each point from 1 to 9). Any additional outcomes listed will be reviewed by the researchers (AME and FPD). If they are deemed to represent a new outcome based on this review, they will be included for round 2 and the SAG will be consulted to review if appropriate. All outcomes will be carried forward to round 2. Of note, continuation to round 2 will be considered based on the response to round 1. If a low number of responders (<10) is observed for one or more stakeholder groups, the Delphi protocol for future rounds will be reviewed and revised. If there is only one stakeholder group with a small number of respondents, then consideration will be given to grouping with another stakeholder group following consultation with the SAG to ensure appropriateness of grouping.

#### Delphi study round 2

Participants who respond to round 1 will be forwarded the round 2 questionnaire and asked to complete it online. Each participant will be presented with the number of respondents and distribution of scores for each outcome per stakeholder group. They will also be shown their score from round 1, asked to consider responses from the other members of the stakeholder groups and invited to rescore the outcome.

#### Analysis of round 2

The results of round 2 will be summarised using descriptive statistics. The number of participants taking part will be recorded. For each outcome, the number of participants who have scored the outcome and the distribution of scores will be noted. All outcomes that have a median score ≥4 for any group will be carried forward to round 3. Participants who completed rounds 1 and 2 will be invited to complete round 3.

#### Delphi study round 3

Participants who respond to rounds 1 and 2 will be forwarded the round 3 questionnaire and asked to complete it online. Again, each participant will be presented with the number of respondents and distribution of scores for each outcome for their particular stakeholder group. They will also be shown their score from round 2, asked to consider responses of other members of the stakeholder group and asked to rescore the outcome.

#### Analysis of round 3

The results of round 3 will be summarised using descriptive statistics. The number of participants taking part will be recorded. For each outcome, the number of participants who have scored the outcome and the distribution of scores will be noted. Each outcome will be classified as ‘consensus in’, ‘consensus out’ or ‘no consensus’ according to the classifications in Table 1. Although these cutoffs are subjective, they have previously been described in a COS protocol and are prespecified to reduce researcher bias [12]. The results of this process will be brought forward to the consensus group meeting.

**Table 1:** Definitions of consensus^a^

Classification	Definition
Consensus in	≥70 % participants scoring as 7–9 and <15 % participants scoring as 1–3
Consensus out	≥70 % participants scoring as 1–3 and <15 % participants scoring as 7–9
No consensus	Anything else

^a^Developed according to the Management of Otitis Media with Effusion in Cleft Palate protocol [12]

#### Consensus group meeting

##### Methods

This final phase will involve a face-to-face meeting with key stakeholders. The meeting participants will be sampled purposively to ensure a range of views of patients, clinicians and researchers with expertise in diabetes care. The sample will be drawn from among those who completed all rounds of the Delphi study. The results obtained from each round of the Delphi survey will be presented. Review of the responses from round 3 of the Delphi exercise will inform the structure and content of the meeting. The objective of the consensus meeting is to discuss outcomes about which there was disagreement in round 3 of the Delphi study and to validate and agree on a list of final outcomes which will constitute the COS.

#### Participants

The consensus group will include, at a minimum, two representatives from obstetrics, endocrinology, midwifery, diabetes specialist nursing and dietetics; at least three service users; and members of the SAG. A half-day meeting is planned and to achieve effective consensus the facilitator will ensure that the meeting is collaborative, cooperative, egalitarian, inclusive and participatory.

## Discussion

There is currently no COS for studies assessing the effectiveness of prepregnancy care for women with pregestational diabetes mellitus. The aim of development of such a COS in this clinical area is to improve the interpretation and comparison of future studies while reducing the risk of outcome reporting bias. We will involve key stakeholders and use recognised techniques to ensure that the ensuing COS is suitable and well accepted in future research.

## Trial status

The study plan is to complete the systematic review by January 2016 and development of the core outcome set by November 2016.

## Additional file

Additional file 1:
**Systematic review search strategies.** This file outlines the detailed search strategies and time limits applied to each of the databases used in the systematic review. (DOCX 64 kb)

## Abbreviations

COS: Core Outcome Set
HbA1c: glycosylated haemoglobin A1c
SAG: study advisory group
