# Supplementary material for: Effectiveness of prepregnancy care for women with pregestational diabetes mellitus: protocol for a systematic review of the literature and identification of a core outcomes set using a Delphi survey
Source: Trials. 2015 Aug 14;16:356. doi: 10.1186/s13063-015-0894-8 (PMC4536746; doi:10.1186/s13063-015-0894-8)
Supplement: Additional file 1: — Systematic review search strategies. This file outlines the detailed search strategies and time limits applied to each of the databases used in the systematic review. (DOCX 64 kb) [file 13063_2015_894_MOESM1_ESM.docx]

**Additional File 1: Systematic review search strategies.**

**Cochrane Library**

1989 – 29/04/2015

#1. “prepregnancy”:ti,ab,kw (Word variations have been searched) 75

#2. “pre-pregnancy” :ti,ab,kw (Word variations have been searched) 68

#3. “pre-conception” :ti,ab,kw (Word variations have been searched) 9

#4. “preconception” :ti,ab,kw (Word variations have been searched) 126

#5. #1 or #2 or #3 or #4 270

#6. “diabetes” :ti,ab,kw (Word variations have been searched) 29,331

#7. #5 and #6 65

**Web of Science**

1998 – 29/04/2015

#1. TOPIC: (type 1 diabetes) 86,057

#2. TOPIC: (type 2 diabetes) 116,204

#3. #2 or #1 142,451

#4. TOPIC: (prepregnancy care) 431

#5. TOPIC: (pre-pregnancy care) 308

#6. TOPIC: (pre-conception care) 101

#7. TOPIC: (preconception care) 918

#8. #7 OR #6 OR #5 OR #4 1,593

#9. #8 AND #3 171

**EMBASE**

1990 – 29/04/2015

('type 1 diabetes'/exp or 'type 1 diabetes' or 'type 2 diabetes'/exp or 'type 2 diabetes') and

(('prepregnancy care'/exp or 'prepregnancy care' or 'preconception care'/exp or 'preconception care') or ('pre conception' and care) or ('pre pregnancy' and care)) and

[female]/lim and [humans]/lim

403

**CINAHL**

1988 – 29/04/2015

S1 type 2 diabetes mellitus OR type 1 diabetes mellitus 51,297

S2 prepregnancy care or preconception care 1,232

S3 pre-pregnancy care or pre-conception care 50

S4 preconception or pre-conception 871

S5 prepregnancy or pre-pregnancy 2,173

S6 S2 OR S3 OR S4 OR S5 2,598

S7 S1 AND S6 141

**MEDLINE**

1946 – 29/04/2015

1 Diabetes Mellitus, Type 1/ or Diabetes Mellitus, Type 2/ or Diabetes Mellitus/ (228389)

2 pre-pregnancy.mp. (1494)

3 prepregnancy.mp. (1886)

4 Preconception Care/ or pre-conception.mp. (1676)

5 pre-conception.mp. (243)

6 2 or 3 or 4 or 5 (4868)

7 1 and 6 (315)
